# Oxidative Stress and Replication-Independent DNA Breakage Induced by Arsenic in *Saccharomyces cerevisiae*

**DOI:** 10.1371/journal.pgen.1003640

**Published:** 2013-07-25

**Authors:** Ireneusz Litwin, Tomasz Bocer, Dorota Dziadkowiec, Robert Wysocki

**Affiliations:** 1 Institute of Experimental Biology, University of Wroclaw, Wroclaw, Poland; 2 Department of Genetics, Institute of Applied Biotechnology and Basic Sciences, University of Rzeszow, Kolbuszowa, Poland; 3 Faculty of Biotechnology, University of Wroclaw, Wroclaw, Poland; University of Washington, United States of America

## Abstract

Arsenic is a well-established human carcinogen of poorly understood mechanism of genotoxicity. It is generally accepted that arsenic acts indirectly by generating oxidative DNA damage that can be converted to replication-dependent DNA double-strand breaks (DSBs), as well as by interfering with DNA repair pathways and DNA methylation. Here we show that in budding yeast arsenic also causes replication and transcription-independent DSBs in all phases of the cell cycle, suggesting a direct genotoxic mode of arsenic action. This is accompanied by DNA damage checkpoint activation resulting in cell cycle delays in S and G2/M phases in wild type cells. In G1 phase, arsenic activates DNA damage response only in the absence of the Yku70–Yku80 complex which normally binds to DNA ends and inhibits resection of DSBs. This strongly indicates that DSBs are produced by arsenic in G1 but DNA ends are protected by Yku70–Yku80 and thus invisible for the checkpoint response. Arsenic-induced DSBs are processed by homologous recombination (HR), as shown by Rfa1 and Rad52 nuclear foci formation and requirement of HR proteins for cell survival during arsenic exposure. We show further that arsenic greatly sensitizes yeast to phleomycin as simultaneous treatment results in profound accumulation of DSBs. Importantly, we observed a similar response in fission yeast *Schizosaccharomyces pombe*, suggesting that the mechanisms of As(III) genotoxicity may be conserved in other organisms.

## Author Summary

Arsenic is a highly toxic compound which causes several types of cancer in humans. However, precise mechanisms of arsenic carcinogenesis remain elusive and are still a matter of debate. For example, the oxidative stress theory of arsenic proposes that arsenic generates reactive oxygen species producing oxidative DNA damage that can be converted to DNA double-strand breaks (DSBs) during replication. Using budding yeast as a model organism, we show that arsenic is able to induce DSBs in the absence of transcription, replication and pronounced oxidative stress. Importantly, we also demonstrate that arsenic greatly enhances cytotoxic activity of antitumor drug phleomycin, as evidenced by increased sensitivity and DNA fragmentation visible upon co-treatment. Our work suggests that arsenic acts as a direct inducer of DNA breaks and could be potentially used with other anticancer drugs, like phleomycin-related bleomycin, as a new combinatory therapy to treat cancers that poorly respond to these drugs. Additionally, since in many countries millions of people are exposed to high doses of arsenic in drinking water, we believe that our findings about genotoxicity of arsenic are important not only to geneticists but also to the general public.

## Introduction

Arsenic is a toxic element ubiquitously present in the environment. Carcinogenic properties of arsenic have been known for a long time and chronic exposure to arsenic in humans has been implicated in numerous types of cancer, including skin, lung, liver, kidney and bladder cancer [1]. On the other hand, due to its cytotoxic properties arsenic is successfully used as antileukemic drug [2] and in the treatment of tropical diseases caused by the protozoan parasites [3]. Since exposure of millions of people to high doses of arsenic in drinking water constitutes a serious health problem [4] and because of increasing use of arsenic as therapeutic agent [5], it is of great importance to elucidate the mechanisms of arsenic toxicity and tolerance.

Up to now, several mechanisms have been proposed to explain carcinogenicity of arsenic, including increased formation of reactive oxygen species (ROS) causing oxidative DNA damage such as single-strand breaks (SSBs) that can be processed to double-strand breaks (DSBs) during replication, inhibition of DNA repair and enhancing mutagenicity and carcinogenicity of other factors, like UV light, global changes in DNA methylation and histone modifications and spindle disruption [6]. In human cell lines exposed to arsenic an accumulation of oxidative DNA damage in the form of 8-hydroxy-2′-deoxyguanosine (8-OHdG) has been shown, which is reversed by addition of antioxidants [7]. On the other hand, inhibition of mRNA synthesis of key base excision repair (BER) enzymes, polymerase beta, AP endonuclease, DNA ligase I and III, as well as enzymatic activity of DNA ligases, have also been observed in the presence of arsenite [As(III)] [8], [9]. These results imply that arsenic increases levels of oxidative stress and at the same time inhibits repair of oxidative DNA damage by BER. Decreased expression of nucleotide excision repair (NER) genes, like ERCC1, XPF and XPA, has been detected in the cells isolated from humans exposed to arsenic in drinking water [10]. Recently it has been reported that poly(ADP-ribose) polymerase 1 (PARP-1) is inhibited by As(III) and proposed that As(III) binding to a zinc finger domain instead of zinc is responsible for inactivation of the PARP-1 protein [11]. In support of this notion, Zhou et al. [12] have just shown that As(III) interacts selectively with zinc finger motifs. Thus both As(III)-induced decrease of BER and NER enzyme expression and inhibition of poly(ADP-ribosyl)ation by As(III) is a likely mechanism for co-carcinogenic activities of arsenic in UV light-induced skin carcinogenesis. Impairment of BER and NER action by As(III) likely results in accumulation of SSBs and other types of DNA lesions which perturb replication fork progression leading to fork collapse and generation of DSBs. Indeed, it has been recently demonstrated in human cell lines that As(III) induces replication-dependent DSBs which are repaired by HR [13]. Additionally, in As(III)-treated cells chromosome aberrations and formation of micronuclei are often observed [6]. As(III) shows high affinity to tubulin and inhibits its polymerization, thus likely contributing to spindle formation and chromosome segregation defects [14], [15]. However, none of above mechanisms has been directly linked to carcinogenesis, while the genotoxic potential of arsenic is still the subject under debate.

The yeast *Saccharomyces cerevisiae* proved to be an excellent model organism to study the mechanisms of action of various DNA damaging agents. It has been reported that As(III) delays the budding yeast cell cycle in all phases [16] and induces phosphorylation of the Rad53 checkpoint kinase (CHK2 in humans) [17]. Importantly, several genome-wide screens have revealed that deletion of yeast genes encoding proteins involved in sensing and repairing of DNA damage, e.g. genes for the Mre11-Rad50-Xrs2 complex (Mre11-Rad50-Nbs1 in humans) [18]–[21], Yku70 involved in non-homologous end joining (NHEJ) [19] and homologous recombination (HR) proteins Rad51 [21], Rad57 [21] and Rad52 [18]–[22], resulted in increased sensitivity to As(III). However, the role of DNA damage response in cell cycle regulation and genomic integrity during As(III) stress as well as the mechanisms of As(III) genotoxicity have never been investigated in greater detail. The purpose of this study was to identify the types of DNA damage generated by As(III) in budding yeast and DNA repair pathways involved in removing such lesions depending on the cell cycle stage. We also sought to investigate the role of DNA damage checkpoints in surviving exposure to As(III).

We have collected several lines of evidence suggesting that the effect of As(III) on DNA is more complex than previously thought and involves a direct generation of DSBs throughout the cell cycle in addition to oxidative and replication-associated DNA damage. We also found that budding and fission yeast simultaneously exposed to As(III) and the DSB-inducing drug phleomycin suffer from a massive chromosome breakage leading to cell death. This would suggest that both drugs could be combined to develop more efficacious anticancer therapies.

## Results

### DNA Damage Checkpoint in Response to Arsenic

**Figure 1:**
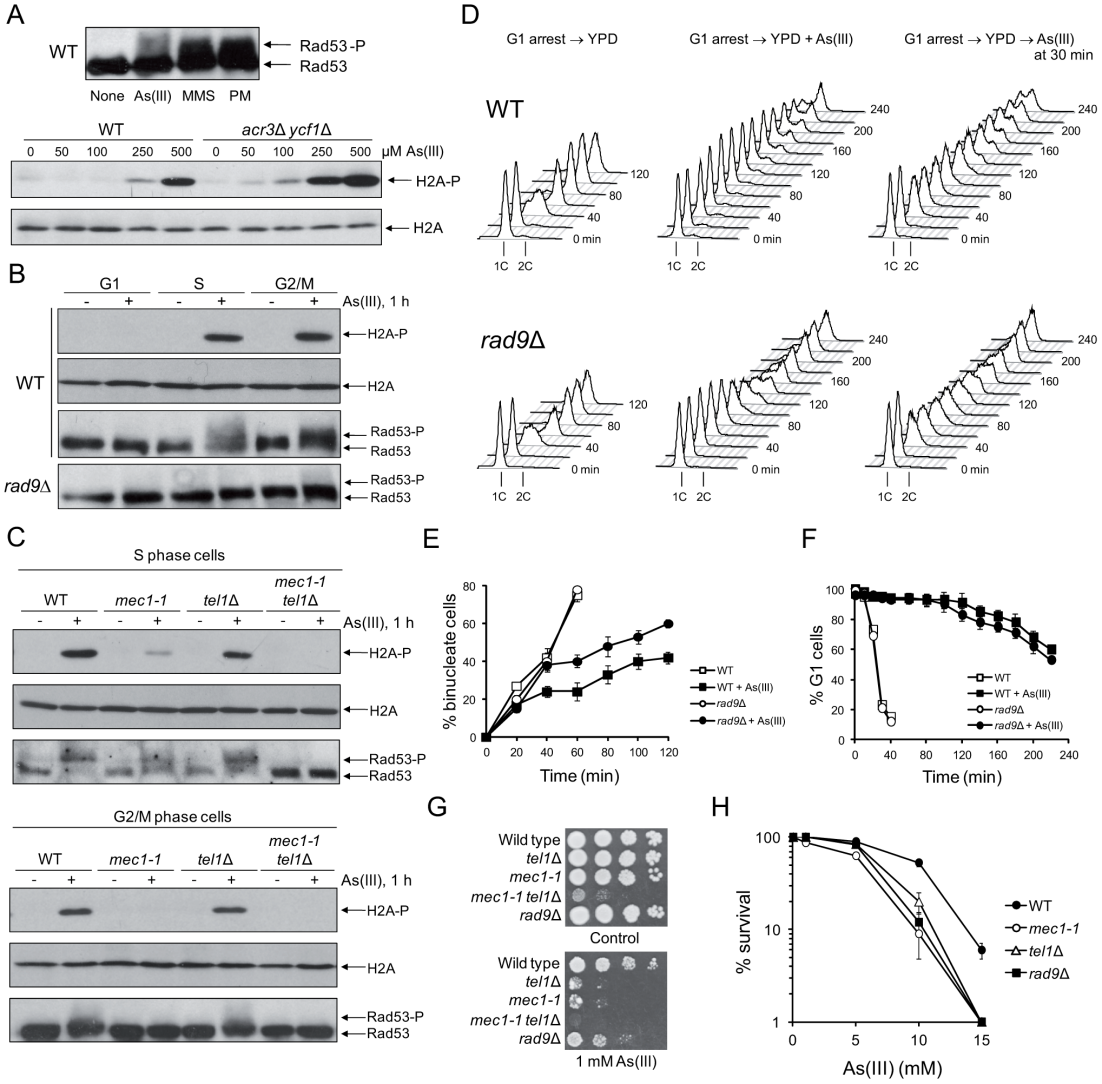
Cell cycle phase-dependent activation of DNA damage checkpoints by As(III) in budding yeast. (A) As(III) triggers activation of DNA damage response in yeast. Exponentially growing wild type (WT) cells were treated with 0.5 mM sodium arsenite [As(III)], 0.01% methyl methanesulfonate (MMS) or 5 µg/ml phleomycin (PM) for 1 h before protein extraction (upper panel). WT and the *acr3*Δ *ycf1*Δ mutant lacking arsenic detoxification transporters were exposed to indicated concentrations As(III) for 1 h (lower panel). (B) As(III) promotes histone H2A phosphorylation and Rad9-dependent hyperphosphorylation of Rad53 in S and G2/M but not in G1 phase. Cells were treated with 0.5 mM As(III) for 1 h. (A–B) Total protein extracts were analyzed by Western blot with anti-Rad53 antibodies to detect unmodified (Rad53) and hyperphosphorylated (Rad53-P) forms of the checkpoint effector kinase Rad53 as well as with anti-phospho-S129 H2A antibodies and anti-H2A antibodies as a loading control. (C) As(III) induces Mec1 and Tel1-dependent activation of DNA damage checkpoints. Experiments were performed as in (B). (D) Flow cytometry analysis of cell cycle progression during 0.5 mM As(III) treatment reveals a partial lack of DNA synthesis slowing in *rad9*Δ compared to WT. (E) Duration of G2/M checkpoint arrest is partially dependent on DNA damage signalling pathway during exposure to As(III). Cells were synchronized in G2/M with nocodazole and released in fresh media in the presence or absence of 0.5 mM As(III). (F) G1/S transition delay in the presence of 0.5 mM As(III) is not maintained by DNA damage checkpoint as shown by the α-factor-nocodazole trap assay. (G, H) DNA damage checkpoint mutants showed increased sensitivity to As(III). Serial dilutions of indicated strains were plated on rich media in the presence or absence of As(III) at 30°C and photographed after 3 days (G) or cells were exposed to indicated concentrations of As(III) for 6 h in liquid minimal media before plating on YPD plates to score viability (H). (E,F,H) Results are shown as means with standard deviations from three independent experiments.

To study the role of DNA damage checkpoints during arsenic stress in *S. cerevisiae*, we first compared the phosphorylation level of the checkpoint effector kinase Rad53 (CHK2 in humans) in wild type cells in response to non-growth inhibitory concentration of 0.5 mM sodium arsenite [As(III)] and other DNA damaging agents, like the DSB-inducing drug phleomycin (PM) and the DNA alkylating agent methyl methanesulfonate (MMS). Rad53 is hyperphosphorylated in response to DSBs in all phases of cell cycle as well as during replication stress as a result of exposition of single strand DNA (ssDNA) gaps [23]–[25]. As expected, we found high levels of slow-migrating hyperphosphorylated form of Rad53 in response to PM and MMS (Figure 1A). In agreement with a previous report [17], 1 h treatment with 0.5 mM As(III) triggered moderate activation of Rad53 (Figure 1A). Histone H2A (yeast H2AX) phosphorylation at S129 is considered to be a sensitive marker of both DSBs and replication fork stalling [26]–[29]. We found that 0.5 mM As(III) promotes high-level phosphorylation of histone H2A (Figure 1A). However, histone H2A activation was not detected with concentrations lower than 0.25 mM As(III). In human cells H2AX phosphorylation is induced at 10–100-fold lower concentrations of As(III) [13] but mammalian cells are much more sensitive to As(III) than yeast due to the lack of metalloid-specific detoxification transport systems [30]. Thus, we also checked the level of histone H2A phosphorylation in the *acr3*Δ *ycf1*Δ double mutant devoid of As(III) transporters [30] and found a dose-dependent increase of histone H2A phosphorylation starting from 0.05 mM As(III) (Figure 1A).

It has been suggested that in mammalian cells As(III) generates only replication-dependent DSBs [13]. Thus, we monitored As(III)-induced activation of H2A and Rad53 in various phases of the yeast cell cycle. Wild type yeast were synchronized in G1 by 5 µM α-factor and treated with 0.5 mM As(III) for 1 h or left untreated in the presence of α-factor to prevent entering S phase. Alternatively, G1-synchronized cells were released in the absence of α-factor to allow progression into S phase and after 30 min were exposed to 0.5 mM As(III) for 1 h. G2/M-arrested cells were obtained by incubation with 15 µM nocodazole followed by 1 h exposure to 0.5 mM As(III) in the presence of nocodazole to inhibit completion of mitosis. We found that histone H2A, and Rad53 are phosphorylated in S and G2/M cells exposed to As(III) but no DNA damage response activation was observed in G1-synchronized cells (Figure 1B). Importantly, in the absence of the checkpoint adaptor/mediator protein Rad9 (53BP1, BRCA1 or MDC1 in humans) Rad53 was not hyperphosphorylated indicating that As(III) induces the classical DNA damage response (Figure 1B).

In yeast activation of DNA damage signalling cascades requires two sensor kinases Mec1 and Tel1 (ATR and ATM in humans), which belong to the phosphoinositide 3-kinase-related kinases (PIKKs) family [23], [31]. Tel1 is involved in sensing DSBs, while Mec1 is activated by the RPA-coated ssDNA structures, which are present at stalled replication forks but are also formed as a result of DSB resection [31]. To determine the roles of Mec1 and Tel1 in As(III)-induced activation of DNA damage checkpoint response, we investigated phosphorylation level of histone H2A and Rad53 in *mec1-1* and *tel1*Δ mutants synchronized in S and G2/M phases. We found that histone H2A and Rad53 activation in G2/M cells was fully dependent on the Mec1 kinase as no phosphorylated forms of both H2A and Rad52 were detected in *mec1-1* cells treated with As(III), while in the *tel1*Δ mutant activation of H2A and Rad53 was at the wild type level (Figure 1C). A similar response was observed in S phase, however, we detected a residual level of phosphorylated H2A and Rad53 in *mec1-1* cells but not in the double *mec1-1 tel1*Δ mutant (Figure 1C). This indicates that Mec1 is a major sensor kinase responsible for As(III)-induced activation of DNA damage signalling with a minor involvement of Tel1 kinase in S phase. Interestingly, DSB-inducer PM induces a similar pattern of DNA damage response activation in S and G2/M phases [32], while hydrogen peroxide (H_2_O_2_) and MMS trigger DNA damage response exclusively in S phase [33], [34]. This might suggest that As(III) is capable of producing both replication-dependent and independent DSBs.

Activation of DNA damage response often leads to cell cycle delay to allow time for DNA repair [31]. Thus, we compared cell cycle progression of wild type cells and the checkpoint-defective *rad9*Δ mutant upon exposure to As(III). In the presence of As(III), cells lacking Rad9 progressed faster through S phase than wild type cells as seen by flow cytometry analysis (Figure 1D) and showed G2/M checkpoint arrest defect measured by counting binucleate cells which completed mitosis (Figure 1E). Analysis of G1/S transition by the α-factor-nocodazole trap assay revealed DNA damage checkpoint-independent arrest in G1 during As(III) treatment (Figure 1F), which is in agreement with the lack of Rad53 and histone H2A phosphorylation in this phase (Figure 1B).

Finally, we analyzed whether activation of DNA damage checkpoints affects cell viability in the presence of As(III). All tested checkpoint-defective mutants showed reduced growth on 1 mM As(III)-containing solid media (Figure 1G). We also checked survival of these mutants during short-term acute exposure to high concentrations of As(III) (Figure 1H) and confirmed a significant role of Mec1-, Tel1- and Rad9-dependent DNA damage checkpoint activation in coping with As(III) toxicity.

### The Role of Oxidative Stress and Replication in As(III)-Induced DNA Damage

**Figure 2:**
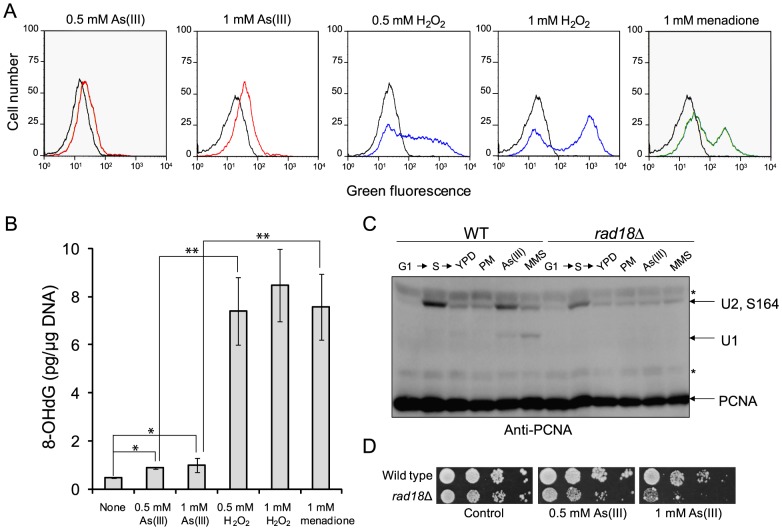
As(III) treatment induces low level of oxidative stress and replication-associated DNA damage. (A) Wild type cells were exposed to indicated concentrations of As(III) (red line), H_2_O_2_ (blue line) and menadione (green line) for 2 h or left untreated (black line). Levels of ROS were determined by measuring green fluorescence of rhodamine 123 (RH3) formed by oxidation of dihydrorhodamine 123 (DHR123) using flow cytometry. (B) Oxidative DNA damage in the form of 8-hydroxy-2′-deoxyguanosine (8-OHdG) induced by indicated concentrations of As(III), H_2_O_2_ and menadione after 2 h exposure. Standard deviations are derived from three independent experiments (*p<0.01, **p<0.001; Student's *t*-test). (C) PCNA ubiquitylation is slightly increased in response to As(III). Wild type (WT) and *rad18*Δ cells were synchronized in G1 with α-factor (G1) and released in fresh medium for 30 min to reach middle S phase (S). Then cells were left untreated (YPD) or treated with either 5 µg/ml phleomycin (PM), 0.5 mM As(III) or 0.01% methanesulfonate (MMS) for 1 h followed by protein extraction. Analysis of PCNA modifications was performed with total protein extracts and anti-PCNA antibodies. Bands corresponding to monoubiquitylated (U1), polyubiquitylated (U2) and sumoylated (S164) forms of PCNA are indicated. Non-specific bands are depicted by asterisks. (D) Cells lacking the Rad18 ubiquitin ligase involved in PCNA monoubiquitylation exhibited increased sensitivity to As(III). Serial dilutions of indicated strains were plated on rich media in the presence or absence of As(III) at 30°C and photographed after 2 days.

A pronounced activation of DNA damage checkpoint in S phase and DNA synthesis completion delay in the presence As(III) (Figure 1) suggests that As(III) exposure leads to oxidative and replication-associated DNA damage as it is observed in mammalian cells [7], [13]. To test this directly, we first checked whether As(III) induces formation of ROS by measuring oxidation of dihydrorhodamine 123 (DHR123) to fluorescent product rhodamine 123 (R123) by flow cytometry (Figure 2A). Levels of ROS were monitored in *S. cerevisiae* cells at several time-points during 2 h exposure in the presence of As(III) as well as H_2_O_2_ or menadione used as positive controls for oxidative stress. We observed a gradual accumulation of ROS in each treatment with a maximum level at 2 h time point shown in Figure 2A. However, exposure to As(III) resulted only in a slight increase of R123 green fluorescence indicating the presence of low levels of As(III)-induced ROS. In contrast, H_2_O_2_ and menadione treatments led to a massive accumulation of R123. Consequently, exposure of *S. cerevisiae* cells to As(III) resulted in 2-fold increase of oxidative DNA damage in the form of 8-OHdG, while H_2_O_2_ and menadione treatments caused 8.5-fold increase of 8-OHdG production compared to control conditions (Figure 2B). The data suggest that in budding yeast As(III) is a weak inducer of oxidative stress and thus produces low levels of oxidative DNA damage.

Next, we asked whether As(III)-induced oxidation of DNA leads to replication perturbations which can be monitored by detecting post-translational modifications of proliferating cell nuclear antigen (PCNA), a processivity factor for DNA polymerases and platform for binding other proteins involved in DNA replication and repair [35]. PCNA is sumoylated at K164 during normal S phase to prevent unscheduled recombination events during replication and mono- and polyubiquitylated at the same residue in response to replication fork stalling due to nucleotide depletion or DNA polymerase-blocking lesions [36]. PCNA ubiquitylation is observed in response to hydroxyurea (HU), MMS, UV and H_2_O_2_ treatment but not to campthotecin which causes replication fork collapse or DSB-inducing drugs like bleomycin [37]. Monoubiquitylation of PCNA is mediated by the Rad6 ubiquitin-conjugating (E2) enzyme and the Rad18 ubiquitin ligase (E3) and promotes the error-prone translesion synthesis (TLS) by recruiting TLS polymerases that are able to catalyze DNA synthesis across the damaged template [35]. Monoubiquitylated PCNA can be further polyubiquitylated by E2 Ubc13-Mms22 and E3 Rad5 to trigger an error-free mechanism of DNA damage bypass which engages template switch and recombination proteins [35]. As expected we detected ubiquitylation of PCNA in the presence of MMS used as a positive control of replication stress inducer while no ubiquitylation of PCNA was observed in response to PM exposure or in the *rad18*Δ mutant under any conditions studied (Figure 2C). During exposure to 0.5 mM As(III) in S phase we found a faint band of monoubiquitylated PCNA indicating that cells experience some level of DNA lesions blocking replication (Figure 2C). The presence of polyubiquitylated PCNA was difficult to assess as diubiquitylated and sumoylated forms of PCNA migrate roughly at the same speed in our SDS-PAGE gels. The physiological importance of PCNA ubiquitylation in coping with As(III)-induced replication perturbations is evident in cells lacking the Rad18 ubiquitin ligase which showed increased sensitivity to As(III) (Figure 2D). Interestingly, the *rad18*Δ and *rad6*Δ mutants were also identified as weakly sensitive to As(III) in genome-wide screens [18], [20], [21].

In sum, our results indicate the ability of As(III) to produce oxidative DNA damage, however at relatively low levels compared to H_2_O_2_ or menadione, which may result in replication perturbations manifested by ubiquitylation of PCNA.

### Arsenic-Induced Replication-Independent DNA Breakage

**Figure 3:**
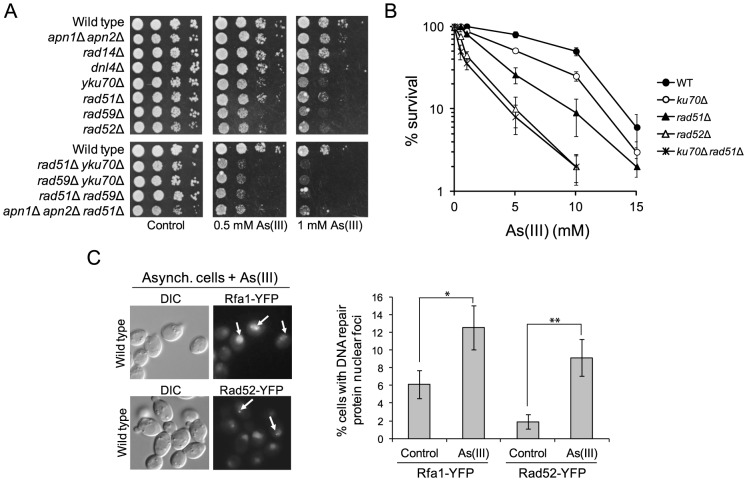
The role of DNA repair pathways in tolerance to As(III) in budding yeast. (A) Homologous recombination and single-strand annealing DNA repair pathways as well as the presence of Yku70 are required for maintaining viability of yeast cells in the presence of As(III). 10-fold serial dilutions of the indicated strains were spotted on rich media that contained either no drug (control) or sodium arsenite [As(III)] and incubated at 30°C for 2 days. (B) As(III)-induced killing of wild type and indicated DNA repair mutants treated with various concentrations of As(III) in minimal media for 6 h. After treatment cells were plated on solid YPD media. The percentage is the ratio of colonies arising after As(III) exposure vs. mock treatment. Results are shown as means with standard deviations from three independent experiments. (C) Homologous recombination DNA repair centers are formed after 1 h treatment with 0.5 mM As(III) as visualized by detection of Rfa1-YFP and Rad52-YFP foci in nuclei with fluorescence microscopy. Standard deviations are derived from three independent experiments (*p<0.05, **p<0.01; Student's *t*-test). DIC, differential interference contrast.

In haploid yeast DSBs are mainly repaired by HR during S and G2/M phases, while NHEJ plays a minor role as this pathway is quite inefficient in re-joining imprecise DNA ends [38]. To test our hypothesis that As(III) is a genotoxic agent that induces DSBs in budding yeast, we investigated whether HR or NHEJ protect yeast against As(III)-induced DNA damage, by comparing viability of wild type and mutant cells devoid of various components of HR pathways (*rad51*Δ, *rad52*Δ, *rad59*Δ) and NHEJ (*yku70*Δ, *dnl4*Δ) in the presence of As(III) (Figure 3A). We found that all HR mutants tested were more sensitive to As(III) than wild type supporting the notion that HR is required for the repair of As(III)-induced DNA damage presumably DSBs and/or ssDNA gaps that form as a result of replication perturbations. Interestingly, cells lacking Yku70 but not the DNA ligase IV Dnl4, showed increased sensitivity to As(III) suggesting a NHEJ-independent role of Yku70/Yku80 complex in tolerance to As(III). Importantly, BER (*apn1*Δ *apn2*Δ) and NER (*rad14*Δ) defective mutants were not sensitive to As(III). However, the triple mutant *apn1*Δ *apn2*Δ *rad51*Δ showed increased sensitivity to As(III) compared to single *rad51*Δ suggesting that As(III) induces some oxidative damage of DNA repaired by BER, with the majority of As(III)-induced DNA damage being DSBs repaired by HR (Figure 3A).

To better assess the importance of NHEJ and HR proteins for As(III) tolerance, we compared survival of wild type and single *yku70*Δ, *rad51*Δ, *rad52*Δ mutants as well as the *yku70*Δ *rad51*Δ double mutant after 6 h exposure to high concentrations of As(III) (Figure 3B). The *rad52*Δ and *yku70*Δ *rad51*Δ mutants were most sensitive to As(III). The *rad51*Δ mutant showed intermediate sensitivity to As(III), while *yku70*Δ were the least sensitive. This confirmed the essential role of HR in coping with As(III)-induced DNA damage. To show that As(III)-induced DNA damage is actively repaired by HR we monitored nuclear localization of Rfa1-YFP, the large subunit of ssDNA-binding RPA complex, and the DNA recombinase Rad52-YFP, that both form distinct fluorescence foci representing the DNA repair centres of multiple DSBs [39]. In asynchronously growing cells exposed to 0.5 mM As(III) for 1 h we observed 2–3-fold increase of the number of cells with Rfa1 and Rad52 foci over spontaneous levels (Figure 3C).

**Figure 4:**
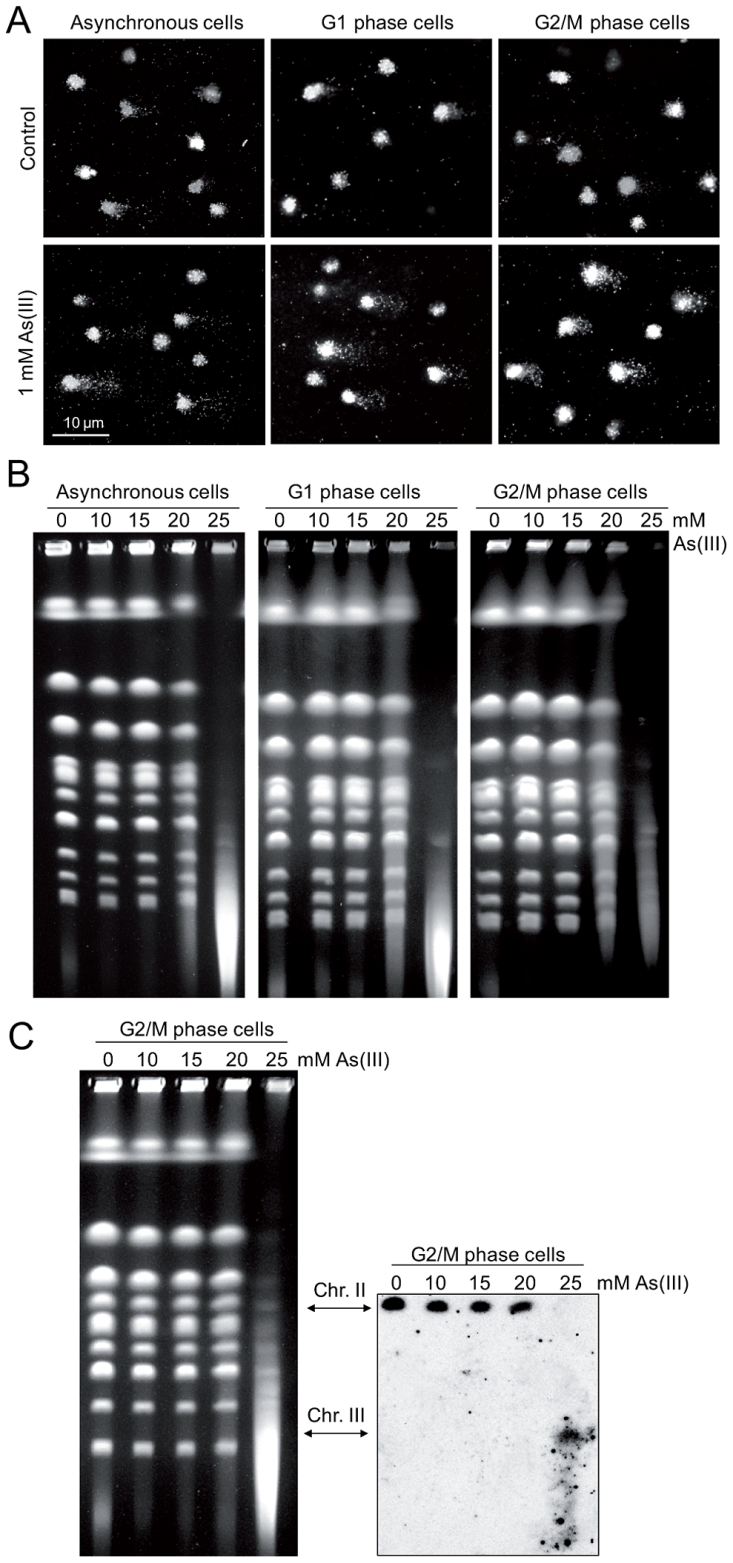
DNA breakage induction by As(III). (A) As(III) induces DNA breakage in all phases of the yeast cell cycle as revealed by the comet assay. Asynchronous, G1 or G2/M-arrested wild type cells (W303-1A) were exposed to 1 mM As(III) for 1 h or left untreated followed by a single-cell gel electrophoresis. Representative images of comets are shown. (B) DSBs induction after As(III) treatment was analyzed by PFGE. Logarithmically growing, G1 and G2/M wild type cells were treated with indicated concentrations of As(III) in minimal media for 6 h and processed for PFGE analysis. In the case of G1 and G2/M-synchronized cells, α-factor or nocodazole were also added during As(III) treatment to maintain cell cycle arrest. (C) As(III)-induced DSBs in G2/M arrested MWJ49 cells containing a circular chromosome III were measured using PFGE followed by Southern hybridization of the shown gel with a *LEU2*-probe to detect chromosome II and III. PFGE experiments were repeated at least two times with similar results and representative images are shown.

To directly demonstrate the presence of As(III)-induced DNA breaks throughout the cell cycle, asynchronous, logarithmically growing (mostly in S phase), G1 and G2/M-synchronized *S. cerevisiae* cells were exposed to 1 mM As(III) for 1 h and analyzed by the comet assay, also known as a single cell gel electrophoresis. This method is routinely used in mammalian cells to measure levels of SSBs and DSBs as visualised by the formation of the comet tail [40]. Recently, the comet assay has been adapted and optimized for yeast cells to detect DNA breakage induced by H_2_O_2_ [41], [42], chemical genotoxins [42] and changes in chromatin organization [43]. A comet head represents an intact DNA, while a comet tail is composed of relaxed DNA loops as a result of DNA damage. However, in contrast to the well-defined mammalian comet tails, the typical yeast comet tails appeared rough, grainy, lumpy, with blobs of various sizes, probably due to less compacted chromatin with fewer heterochromatin domains compared to mammalian chromatin [41]–[43]. The yeast comet assay revealed that As(III) induces DNA breaks independently of the cell cycle phase (Table 1 and Figure 4A). The high incidence of the observable DNA damage was detected in logarithmically growing (mostly S phase) cells (16.9%). In contrast, the relatively small number of G2/M cells (6.4%) showed As(III)-induced DNA damage. Interestingly, 14.1% of G1 cells exhibited the presence of DNA breaks after As(III) treatment. The analysis of yeast comets summarized in Table 1 revealed that As(III)-induced comet tails were 2 times longer and contained between 2- and 3-fold more DNA than spontaneous tails. The level of DNA breaks were similar in asynchronous and G1 cells and slightly lower in G2/M cells.

**Table 1:** Analysis of As(III)-induced DNA damage by the comet assay.

Conditions	Cell cycle phase	Comets (%)	Tail DNA (%)	Tail length (µm)	Tail moment (arbitrary units)
	Asynch.	5.4±1.58	6.68±2.19	3.24±1.34	2.03±0.74
Control	G1	2.1±0.76	6.45±2.08	3.02±1.23	1.78±0.76
	G2/M	2.8±0.84	6.52±2.22	2.98±1.33	1.77±0.89
	Asynch.	16.9±4.18	17.13±4.12	6.76±2.13	9.68±3.09
As(III)	G1	14.1±3.71	17.95±5.01	6.99±2.54	10.13±3.32
	G2/M	6.4±2.29	13.11±3.98	5.86±2.18	7.87±2.74

Cells were treated with 1 mM As(III) for 1 h or left untreated for control and subjected to the comet assay. Numbers are averages of three independent experiments with standard deviations. In each experiment 250 randomly chosen comets were analyzed. Asynch., asynchronous cells.

As the alkaline comet assay used in this study does not differentiate between SSBs and DSBs, we performed pulsed-field gel electrophoresis (PFGE) of budding yeast chromosomes isolated from asynchronous, G1- and G2/M-synchronized cells treated with As(III) for 6 h (Figure 4B). The presence of DSBs can be visualized as the disappearance of distinct chromosome bands and accumulation of low molecular weight smear. Considering relative low levels of As(III)-induced DNA breaks measured with the comet assay and previously reported lack of detectable DNA breaks in PFGE in response to 1 mM As(III) [19], we exposed yeast cells to much higher concentrations of As(III). Fragmentation of DNA became evident after treatment with 20 mM As(III) and pronounced degradation of chromosomes was observed in the presence of 25 mM As(III) in all cell cycle phases (Figure 4B). To confirm that As(III)-induced DNA degradation detected with PFGE are caused by replication-independent DSBs, the yeast chromosomes were prepared from G2/M-synchronized MWJ49 strain containing a circular chromosome III which does not enter the gel during PFGE unless is broken into a linear chromosome [44]. The broken chromosome III can be detected by Southern hybridization as a separate band and serves as a measurement of DSB generation. In the presence of 25 mM As(III) we were able to detect the linear form of chromosome III confirming that As(III) induces replication-independent DSBs (Figure 4C).

**Figure 5:**
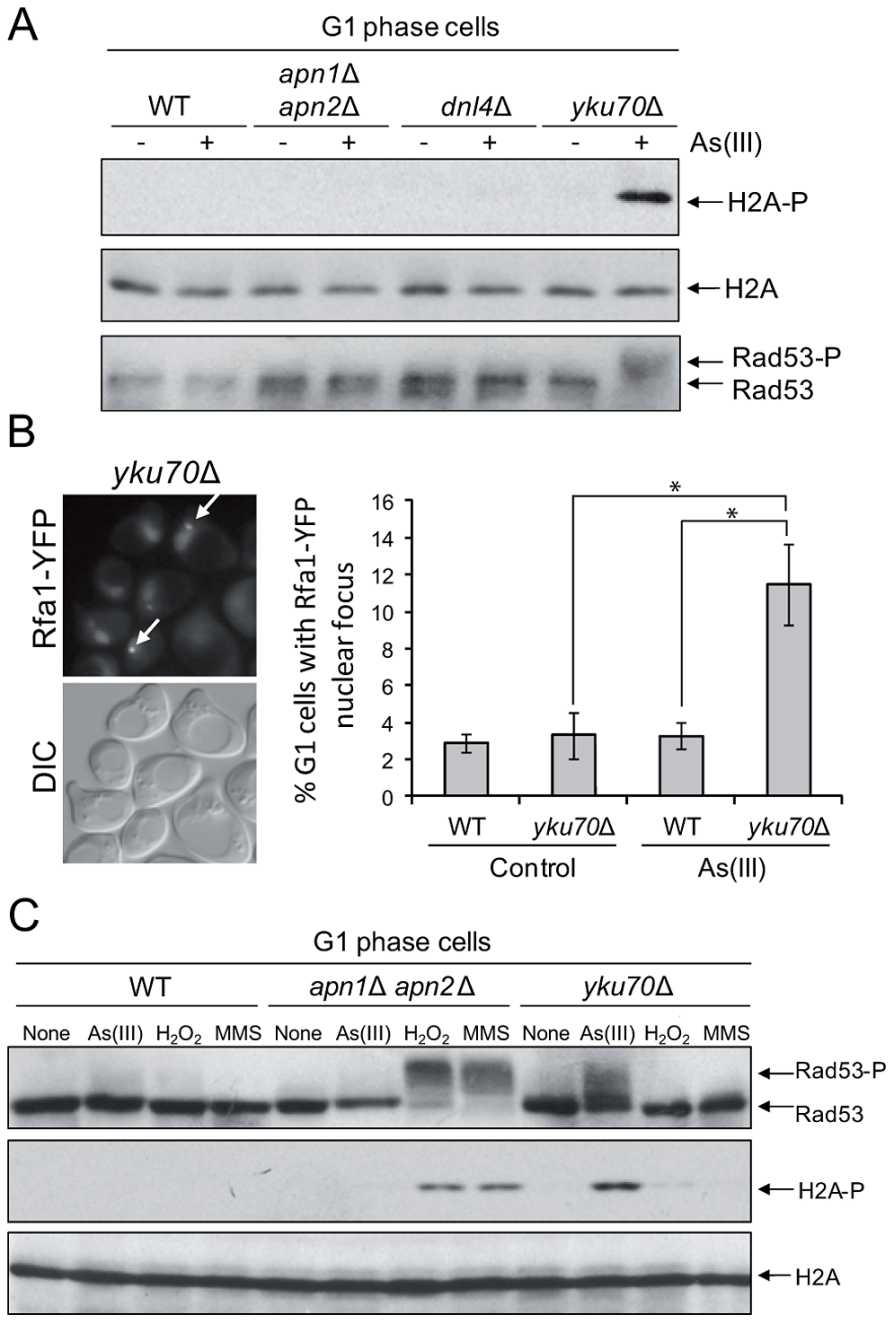
As(III) induces DNA damage checkpoint response in G1 phase in Yku70-deficient cells. (A) Histone H2A and Rad53 phosphorylation induction by As(III) in G1 cells in the absence of Yku70. The indicated strains were synchronized in G1 with α-factor and treated with 0.5 mM As(III) for 1 h followed by protein extraction and Western blot analysis. (B) Accumulation of Rfa1-YFP foci in the G1-synchronized *yku70*Δ strain reveals existence of As(III)-induced DSBs in G1 phase which undergo resection in the absence of Yku70. The representative image of *yku70*Δ cells in G1 phase containing As(III)-induced Rfa1 foci (arrows) is shown. Cell treatment was as in (A). Standard deviations are derived from three independent experiments (*p<0.01; Student's *t*-test). DIC, differential interference contrast. (C) Analysis of DNA damage response activation in G1-synchronized cells devoid of BER (*apn1*Δ *apn2*Δ) or Yku70 reveals that As(III)-induced DNA lesions are different from those generated by H_2_O_2_ or MMS. The indicated strains were exposed to 0.5 mM As(III), 0.5 mM H_2_O_2_ or 0.03% methyl methanesulfonate (MMS) for 1 h and analyzed by western blot.

The presence of As(III)-induced DSBs in G1 revealed by the yeast comet assay and PFGE seemed to contradict our previous observation that As(III) treatment did not lead to phosphorylation of histone H2A and Rad53 in G1 cells (Figure 1) which suggested that As(III)-induced DSBs do not form in this phase of the cell cycle. One possible explanation would be to assume that As(III)-induced DSBs do indeed form but are not resected in G1 and DNA damage signal is not transduced to the checkpoint pathway. It has been shown that oxidative DNA lesions caused by H_2_O_2_ treatment are silently repaired in G1 and G2/M with no activation of Rad53 kinase in these cell cycle phases [33]. In contrast, when BER is compromised by deletion of *APN1* and *APN2* endonuclease genes, H_2_O_2_-induced phosphorylation of Rad53 is observed in all phases of the cell cycle. It has also been reported that endonuclease-induced DSBs are not resected in G1 due to the Yku70–Yku80-dependent but NHEJ-independent protection of DNA ends [45], [46]. In agreement with our hypothesis that in G1 As(III)-induced DSBs are not resected and signalled to DNA damage checkpoint, we demonstrated that the *yku70*Δ mutant, but not the mutants defective in NHEJ (*dnl4*Δ) or BER (*apn1*Δ *apn2*Δ), showed phosphorylation of histone H2A and Rad53 in G1 after As(III) treatment (Figure 5A). Consequently, we detected a markedly elevated level of As(III)-induced Rfa1 foci in the *yku70*Δ mutant in G1 phase indicating the presence of DSBs undergoing 5′ end resection (Figure 5B). As opposed to As(III), exposure to H_2_O_2_ and MMS in G1 phase did not induce the DNA damage response activation in *yku70*Δ mutant, while treatment with these agents but not As(III) led to pronounced levels of H2A and Rad53 phosphorylation in *apn1*Δ *apn2*Δ mutant (Figure 5C). These results strongly suggest that DNA lesions generated by As(III) in G1 are quite specific and different in nature from H_2_O_2_ and MMS-induced DNA damage and thus are not processed by BER.

We have thus demonstrated using several assays that As(III) has the ability to induce DSB in the G1 phase of the cell cycle, i.e. independently of the replication process.

### As(III)-Induced DNA Damage Is Not Coupled to Transcription

**Figure 6:**
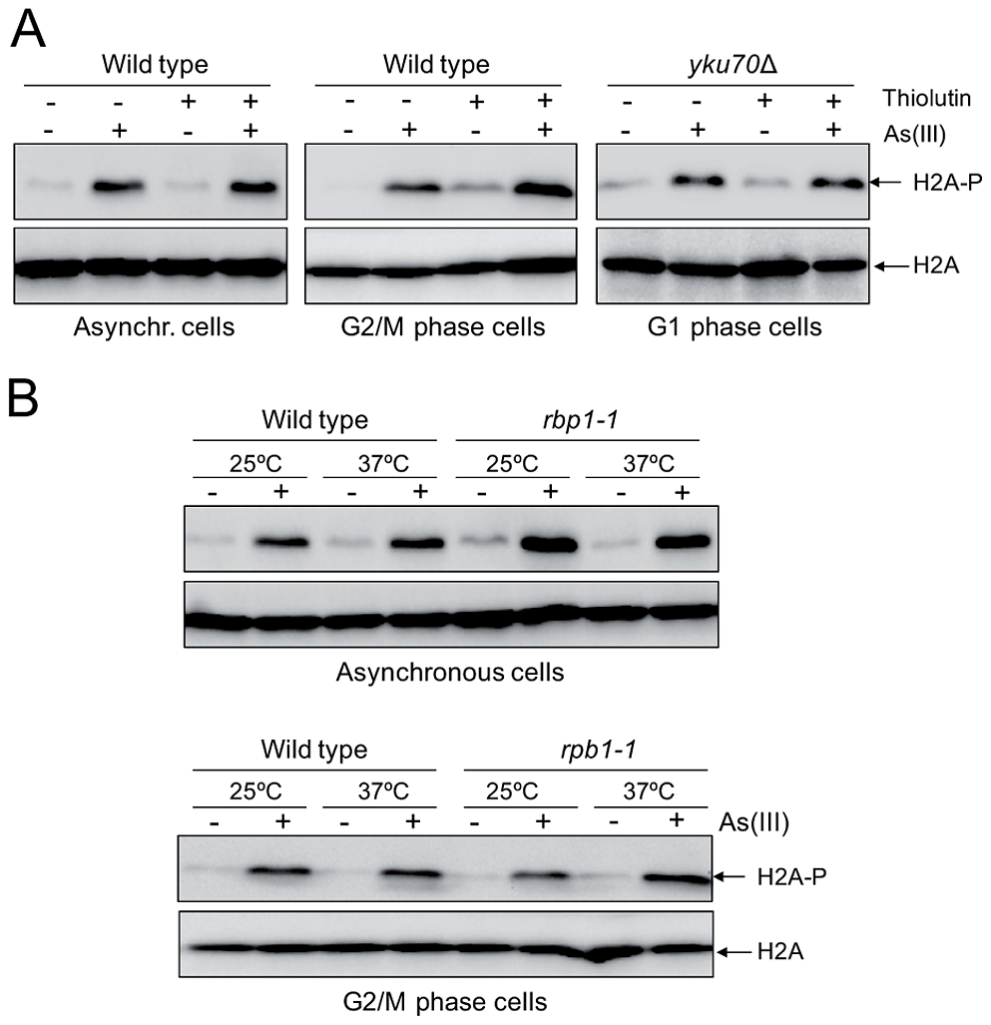
Transcription-independent DNA damage induction by As(III). (A) To shut off transcription 3 µg/ml thiolutin, an inhibitor of RNA polymerases, was added to asynchronous and G2/M phase wild type cells and G1-arrested *yku70*Δ mutant for 1 h and then 0.5 mM As(III) was added to the cells for 1 h followed by protein extraction and western blotting analysis of histone H2A phosphorylation. (B) The *rpb1-1* cells bearing a temperature-sensitive mutation in the catalytic subunit of RNA polymerase II grown at permissive temperature (25°C) were shifted or not to non-permissive temperature (37°C) by adding YPD pre-warmed to 45°C to block transcription and exposed to 0.5 mM As(III) for 1 h. For a control wild type cells were treated in a similar way. Protein extracts were analyzed by western blotting to detect levels of phosphorylated H2A. Total histone H2A was used as a loading control.

Although we showed generation of As(III)-induced DSBs outside S phase indicating that As(III) may act as a direct inducer of DSBs, oxidative DNA damage produced by As(III) might generate transcription-associated DSBs. It has been recently reported that in non-replicating mammalian cells DSBs are formed when transcription is blocked by camptothecin-induced stalling of topoisomerase I (TOP1) cleavage complex which normally removes DNA supercoiling produced by transcription [47]. It has been proposed that under physiological conditions ROS-induced oxidative DNA lesions could also trap TOP1 cleavage complex and generate transcription-associate DSBs [47], [48]. Importantly, addition of chemical inhibitors of RNA polymerase II prevented formation of transcription-linked DSBs in mammalian cells [47]. To determine whether As(III) can induce transcription-dependent DNA damage, we examined phosphorylation of histone H2A at S129 in cells treated with As(III) in the presence of thiolutin which inhibits all three RNA polymerases [49], [50] (Figure 6A). Alternatively, we shut off RNA synthesis by using the *rpb1-1* allele, a temperature-sensitive mutant in the catalytic subunit of RNA polymerase II [50], [51] (Figure 6B). Under both conditions inhibition of transcription did not decrease phosphorylation of histone H2A in both asynchronous and G2/M-arrested wild type as well as in G1-synchronized *yku70*Δ cells treated with As(III) suggesting that As(III)-induced DNA damage is not associated with transcription (Figure 6).

### Arsenic Increases Genotoxicity of Phleomycin

**Figure 7:**
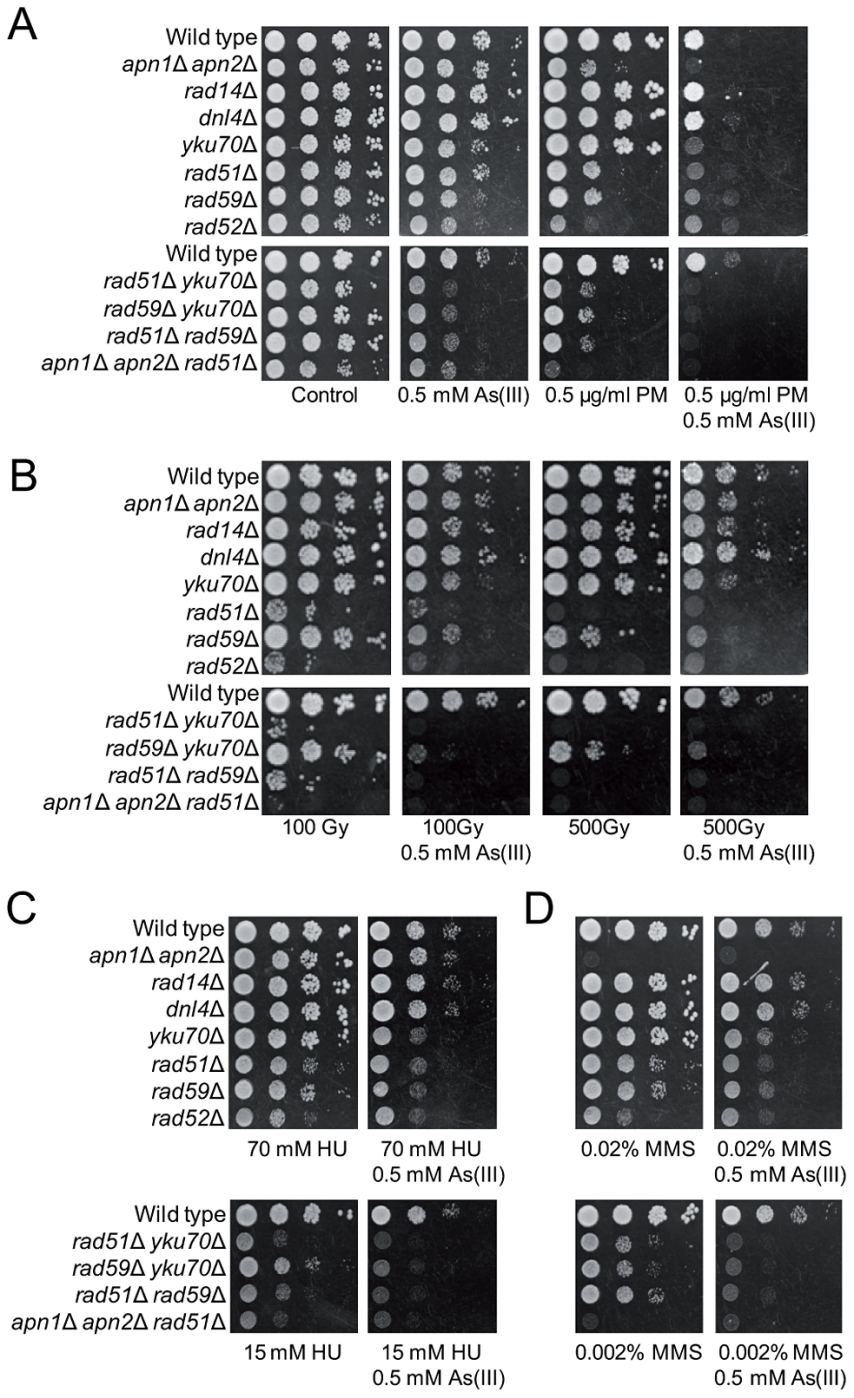
As(III) enhances cytotoxicity of phleomycin. (A) Serial dilutions of the indicated strains were spotted onto control YPD and As(III)-containing YPD plates or media containing phleomycin (PM) with or without As(III). (B) Alternatively, yeast cells were irradiated with various doses of ionizing radiation before plating in the presence or absence As(III). (C, D) Cells were also cultivated on media containing hydroxyurea (HU) (C) or methyl methanesulfonate (MMS) (D) with or without As(III). Cells were incubated at 30°C for 2 days.

It has been reported that As(III) pretreatment can increase the cytotoxic effect of radiomimetic drug bleomycin in Chinese hamster ovary cells, probably by hampering the cellular mechanisms which inactivate bleomycin [52]. In addition, arsenic radiosensitizes cancer cell lines and solid tumors [53]–[55]. Increased death of cancer cells has been explained by elevated ROS production followed by induction of autophagy and apoptosis [53], [54]. To test the cytotoxic effect of DSB-inducing agents combined with As(III) in yeast, growth of wild type cells and DNA repair mutants was tested in the presence of both As(III) and the bleomycin-related agent PM or As(III) and PM alone (Figure 7A). In addition, yeast cells were exposed to 100 Gy and 500 Gy of ionizing radiation (IR) and then plated in the presence or absence of 0.5 mM As(III) (Figure 7B). We found that As(III) profoundly sensitized yeast cells to PM. In the presence of both drugs the growth of all tested yeast strains, both wild type and DNA repair mutants, was strongly inhibited (Figure 7A). In contrast, the combined treatment with As(III) and IR conferred a slight additive effect on growth inhibition of all tested strains indicating that As(III) only weakly increased cytotoxic effect of IR (Figure 7B). Similarly, co-treatment of with As(III) and HU (Figure 7C) or MMS (Figure 7D) moderately increased sensitivity of wild type and DNA repair mutants to these genotoxic drugs. These findings can be interpreted that As(III) specifically enhances ability of PM to generate DNA damage, while in the case of IR, HU and MMS co-treatment we observed the additive cytotoxic effect of two DNA damaging agents acting separately.

**Figure 8:**
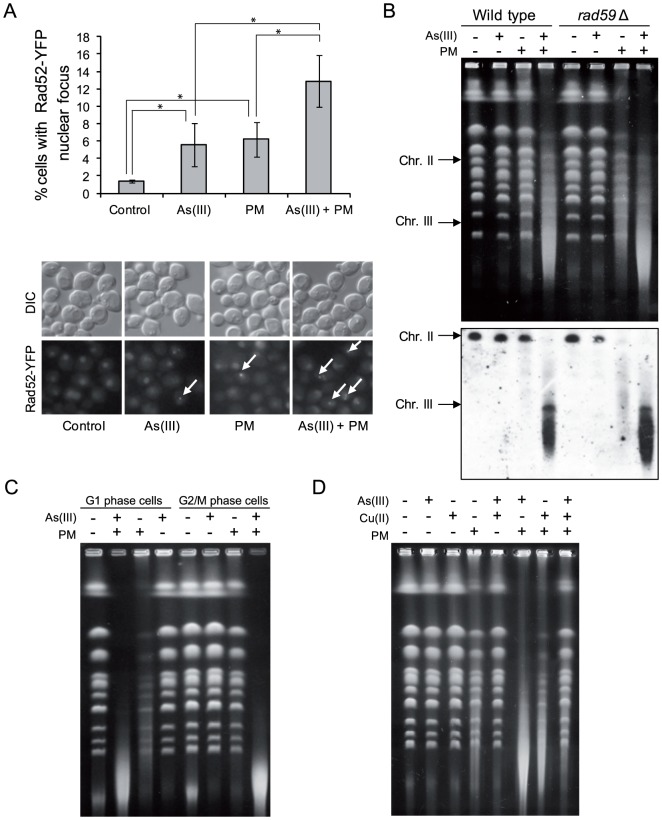
As(III) and phleomycin co-treatment increases formation of DSBs. (A) Increased accumulation of Rad52-YFP nuclear foci in wild type cells after 1 h of 0.5 mM As(III) and 0.5 µg/ml phleomycin (PM) co-treatment. Standard deviations are derived from three independent experiments (*p<0.05; Student's *t*-test). (B) Yeast chromosome breaks in asynchronously growing cells of indicated strains containing a circular chromosome III were measured using PFGE followed by Southern hybridization of the shown gel with a *LEU2*-probe to detect chromosome II and III. (C) DSB induction during As(III) and PM co-treatment in wild type cells synchronized and maintained in G1 or G2/M phase. (B, C) Cells were treated with 5 mM As(III) and 10 µg/ml PM in YPD medium for 4 h. (D) PFGE analysis of *S. cerevisiae* chromosomes isolated from wild type cells exposed to 5 mM As(III), 10 µg/ml PM or 4 mM copper sulfate [Cu(II)] in YPD medium for 4 h. PFGE experiments were repeated at least two times with similar results and representative images are shown.

To investigate whether the enhanced cytotoxicity of combined treatment with As(III) and PM is the result of increased DNA damage in the form of DSBs, we first monitored the formation of Rad52-YFP foci in wild type during As(III) and PM co-treatment and noticed that the number of cells containing Rad52-foci was increased by 2-fold compared to samples treated with a single agent (Figure 8A). This implies that combined treatment with As(III) and PM leads to accumulation of DNA damage which is repaired by HR. In order to show directly that As(III) sensitizes yeast to PM by inducing more DSBs, we performed PFGE and Southern analysis of yeast chromosomes isolated from the MWJ49 yeast strain containing a circular chromosome III [44]. After As(III) or PM treatment we observed no appearance of the linear form of chromosome III (Figure 8B). However, in the case of PM some fragmentation of chromosomes was already evident as a background DNA smear. Interestingly, As(III) and PM co-treatment resulted in a massive breakage of chromosomes and accumulation of the singly broken chromosome III (Figure 8B). Moreover, mutants devoid of *RAD59* gene involved in repair of DSBs by single-strand annealing, which renders yeast cells more sensitive to both As(III) and PM (Figure 7B), accumulated more DNA breaks after single PM and combined treatment (Figure 8B). To show that the observed accumulation of DSBs is the result of direct genotoxicity of As(III) and PM and does not require replication to process SSBs into DSBs, wild type cells were synchronized in G1 or G2/M followed by exposure to As(III) and PM and analyzed by PFGE. This experiment revealed that As(III) and PM co-treatment caused a massive fragmentation of chromosomes also in the absence of replication (Figure 8C).

PM is a copper-chelating peptide which is able to bind to DNA and in the presence of oxygen converts itself to a free radical reactive complex producing oxidized apurinic/apyrimidinic (AP) sites, SSBs and DSBs [56]. In the case of PM-related bleomycin addition of iron ions enhances DNA damage by facilitating contact between bleomycin and DNA as well as by activating oxygen to generate free radicals [57]. Indeed, we showed that extra addition of Cu(II) ions increased PM-induced DNA fragmentation similarly to As(III) (Figure 8D). We also wondered whether the free Cu(II), which is present in the solution of PM added to the cells, is responsible for the observed phenomenon. However, combined exposure to As(III) and Cu(II) in the form of copper sulfate did not result in DNA breakage detectable by PFGE (Figure 8D). Combination of As(III) and Cu(II) did not increase PM-dependent DNA fragmentation but instead inhibited this process, probably as a result of formation of copper arsenite which is insoluble in water (Figure 8D). In sum, these observations suggest a specific interplay between As(III) and PM which leads to cell death as a result of increased DNA fragmentation.

### As(III) Induces DSBs and Enhances Genotoxicity of Phleomycin Also in *Schizosaccharomyces pombe*

**Figure 9:**
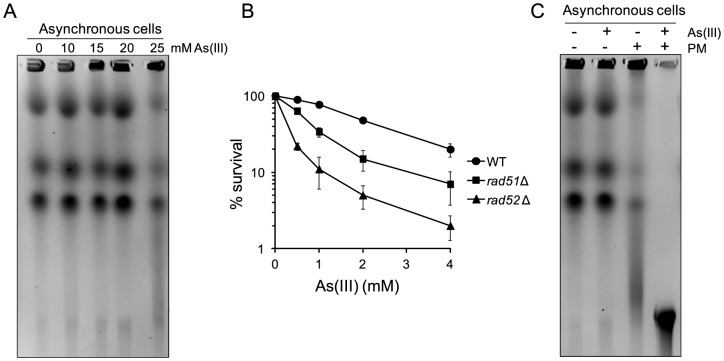
DSB induction by As(III) in *Schizosaccharomyces pombe* and the role of HR in surviving As(III) treatment. (A) DSBs induction after As(III) treatment was analyzed by PFGE. Logarithmically growing (mostly G2/M phase in the case of fission yeast) wild type cells were treated with indicated concentrations of As(III) in minimal medium for 6 h and processed for PFGE analysis. (B) Survival of wild type (WT) and indicated DNA repair mutants treated with various concentrations of As(III) in minimal media for 6 h. After treatment cells were plated on solid YES plates. The percentage is the ratio of colonies arising after As(III) exposure vs. mock treatment. Results are shown as means with standard deviations from three independent experiments. (C) Induction of DSBs by combined treatment with As(III) and PM as revealed by PFGE analysis. Logarithmically growing cells were treated with 5 mM As(III) and 0.25 µg/ml PM in YES medium for 4 h. PFGE experiments were repeated at least two times with similar results and representative images are shown.

To test whether As(III) shows a similar mode of genotocixity in another model organism, we performed the PFGE analysis of chromosomes isolated from a distantly related fission yeast *S. pombe* exposed to increasing concentrations of As(III). As shown in Figure 9A, 25 mM As(III) treatment caused pronounced breakage of fission yeast chromosomes. Moreover, the *S. pombe* mutants devoid of HR proteins Rad51 and Rad52 displayed higher sensitivity to As(III) than wild type (Figure 9B). Consequently, combined treatment with As(III) and PM resulted in a complete degradation of chromosomes (Figure 9C). In sum, this data indicate that the ability of As(III) to induce low levels of DSBs and enhance genotoxicity of PM by generating more DSBs is not restricted to budding yeast. It is thus possible that As(III) may act in a similar way in higher eukaryotes too.

## Discussion

Carcinogenic properties of As(III) are explained in current literature as a result of As(III)-induced accumulation of ROS causing oxidative DNA damage, including SSBs and other types of DNA lesions which perturb replication fork progression leading to fork collapse and generation of DSBs [4], [6], [7], [13]. Besides it has been reported that As(III) inhibits BER and NER pathways further contributing to accumulation of oxidative DNA lesions [8], [9], [13].

Contrary to what was believed previously, we have discovered that As(III) generates not only oxidation of nitrogen bases and SSBs leading to replication-coupled DSBs but also replication-independent DSBs in all phases of the cell cycle. In wild type yeast As(III) induces the DNA damage checkpoint response in both S and G2/M phase (Figure 1) but is also able to phosphorylate histone H2A and Rad53 kinase in G1-arrested cells when the DNA end-binding Yku70–Yku80 complex is absent (Figure 5A). This suggests that As(III) may generate replication-independent DSBs in G1 that are normally bound by Yku70–Yku80 and activate the cell cycle checkpoint only in the *yku70*Δ mutant when the resection of DNA ends is not inhibited. Indeed, we observed formation of Rfa1 nuclear foci in As(III)-treated *yku70*Δ cells arrested in G1 indicating the presence of As(III)-induced DSBs which are resected and coated by Rfa1 (Figure 5B). Accumulation of DNA breaks in all phases of the cell cycle after As(III) treatment was also evident in the comet assay (Figure 4A and Table 1). Since similar number of G1 wild type cells showed the presence of DNA breaks (Table 1) and Rfa1 foci after deletion of *YKU70* which are indicative of DSB resection (Figure 5B), we conclude that As(III)-induced breaks detected by the comet assay mostly represent DSBs (Figure 4A). In agreement with this assumption, we were able to visualize DSBs by PFGE in the absence of replication using high concentrations of As(III) (Figure 4B and 4C). Ability of As(III) to generate DSBs explains increased sensitivity of HR DNA repair mutants to As(III) reported in genome-wide screens [18]–[22] and confirmed in this paper (Figure 3).

It has been shown that replication-associated inducers of DSBs, H_2_O_2_ and MMS, trigger histone H2A and Rad53 phosphorylation exclusively in S phase [33], [58]. Activation of the DNA damage checkpoint proteins during H_2_O_2_ or MMS treatment occurring outside replication is only observed in the absence BER enzymes, Apn1 and Apn2 endonucleases [33], [58] (Figure 5C). Formation of MMS-derived DSBs in G2/M has been recently demonstrated in yeast cells lacking AP endonucleases, probably as a result of accumulation of closely-spaced SSBs on complementary DNA strands [59]. Presence of MMS-induced DSBs in G1 has also been suggested [44]. Our data showing H2A and Rad53 phosphorylation in MMS treated G1-synchronized BER-deficient cells but not in the *yku70*Δ mutant provide indirect *in vivo* evidence about formation of DSBs with ragged ends which are derived from closely-spaced SSBs (Figure 3C). Importantly, As(III) did not induce histone H2A and Rad53 phosphorylation in the *apn1*Δ *apn2*Δ mutant in G1 (Figure 5C) suggesting that majority of As(III)-dependent DNA lesions are not processed by BER and thus do not result from typical oxidative DNA damage. In support of this notion, we showed that As(III) induced only a slight increase of ROS (Figure 2A) and DNA oxidation (Figure 2B) and the *apn1*Δ *apn2*Δ mutant, which is highly sensitive to ROS-inducing agents [33], [60] and MMS [59], [61], [62], is not hypersensitive to As(III) (Figure 3A). Moreover, As(III) only weakly triggers ubiquitylation of PCNA in S phase (Figure 3C) which is a hallmark of replication perturbations caused by UV, MMS and H_2_O_2_-induced DNA damage [36], [37]. In sum, at least in budding yeast our results rule out the possibility that arsenic acts as a powerful DNA oxidizer and suggest that As(III) may directly produce DSBs independently from replication.

However, the question remains how As(III) is able to induce DSBs. Since As(III) generates low level of oxidative damage (Figure 2A and 2B), it is highly improbable that DSBs are formed due to the presence of closely-opposed randomly generated SSBs. Moreover, As(III)-induced DNA damage is not associated with transcription suggesting a direct mode of As(III) genotoxicity (Figure 6). It has been determined by Fourier transform infrared spectroscopy that *in vitro* As(III) is able to bind indirectly to nitrogen bases of DNA [63], [64] but no specific As(III)-DNA or histone-As(III) complexes were detected *in vivo* using radioactive As(III) [65]. Transition metal ions like copper and iron are able to bind to DNA and histones and *in situ* oxidize DNA via Haber-Weiss reactions [66]. However, such action has never been demonstrated for As(III) and we failed to show that As(III) has capacity to cleave plasmid DNA in the presence of H_2_O_2_ (data not shown). Taking into account the presence of As(III)-induced DSBs outside S phase (Figure 4) and independent from transcription (Figure 6), protection of As(III)-induced DSBs by Yku70–Yku80 complex (Figure 5), which preferentially binds to unragged DNA ends [45], lack of enhanced overall production of ROS in the presence of As(III) and high instability of ROS which are unable to diffuse for long distances, we hypothesize that in a similar way as PM As(III) may act in the vicinity of DNA causing *in situ* production of free radicals which sequentially create break in one strand and a directly opposed single-strand break on the complementary strand.

Our finding that As(III) greatly increases the ability of PM to induce DSBs both in *S. cerevisiae* (Figure 8) and *S. pombe* (Figure 9C), could have important potential applications. If this also proves to be the case for bleomycin and human cancer cell lines, a combinatory therapy with As(III) could be envisioned in order to decrease bleomycin therapeutic dose and thus its side effects as well as to treat cancers which are weakly responsive to this drug [57], [67].

## Materials and Methods

### Cell Treatments

The *S. cerevisiae* and *S. pombe* strains used in this study are listed in Table S1. Gene deletions were generated by the PCR-based gene replacement method [68]. The *S. cerevisiae* strains were grown in standard rich YPD medium or minimal (SD) medium supplemented with the required amino acids at 30°C. The *S. pombe* strains were cultivated in standard rich YES medium or Edinburgh minimal medium (EMM) at 30°C. For DNA damage sensitivity tests, cells were grown to logarithmic phase and 10-fold serial dilutions were spotted onto YPD plates containing various concentrations of DNA damaging agents. Alternatively, cells were irradiated at 5 Gy/min with a ^60^Co source before plating. To measure survival of yeast strains after acute treatment with As(III), cells were grown to logarithmic phase and exposed to indicated concentrations of sodium arsenite for 6 h in minimal medium or left untreated. After treatment cells were washed with water, serially diluted and plated on YPD or YES plates. After 3 days of incubation at 30°C colony forming units were counted to determine the number of survived cells. To determine cell cycle phase-dependent response to As(III) treatment, yeast cells were synchronized in G1 phase by 5 µM α-factor or in G2/M phase by 15 µM nocodazole for 2 h followed by exposure to DNA damaging agents in the presence of α-factor or nocodazole to prevent cell cycle progression. Experiments were performed only when at least 90% of cells showed proper cell cycle synchronization confirmed by microscopy observations of unbudded cells showing shmoo projections (G1-synchronized cells) or large-budded cells with a single nuclei (G2/M-synchronized cells) visualized by DAPI (4′,6-diamidino-2-phenylindole) staining.

### Cell Cycle Progression Analysis

Flow cytometry analysis of DNA content was performed as previously described [69]. Briefly, for each time-point 0.5 ml of yeast cells were fixed with 70% ethanol, washed twice with water and incubated for 2 h with 0.25 µg/ml RNase at 50°C followed by 1 h incubation with 1 µg/ml pepsine at 37°C. Next, cells were sonicated, stained with 2.5 µM SYTOX Green for 1 h and analyzed by flow cytometry. The fraction of cells remaining arrested in G1 was determined by the α-factor-nocodazole trap assay [69]. At indicated time-points 0.5 ml of cell culture was washed twice with water and combined with 0.5 ml of YPD medium containing 10 µg/ml α-factor and 30 µg/ml nocodazole and incubated for 90 min at 30°C followed by fixation with 70% ethanol. Next, cells were examined by a light microscope to count cells with shmoo projections (cells remaining arrested in G1) or large-budded cells (post-G1 cells arrested in G2/M). To determine the fraction of post-mitotic cells aliquots were fixed, stained as for flow cytometry, and then observed with an Axio Imager M1 Carl Zeiss epifluorescence microscope (GFP filter set, 40×/0.75 objective) to score the percentage of binucleate large-budded cells. All cell cycle experiments were repeated a minimum of three times.

### Protein Analysis

Total protein extracts were prepared by the trichloroacetic acid method and resolved on SDS-PAGE, blotted onto nitrocellulose filters and probed with anti-Rad53 (Santa Cruz, sc-6749), anti-histone H2A (phospho S129) (Abcam, ab15083), anti-histone H2A (Abcam, ab13923) or anti-PCNA (kindly provided by B.W. Stillman) antibodies. Blotted membranes were stained for total protein with Ponceau S (Sigma) before immunodetection.

### ROS Measurements

To detect increased levels of ROS, wild type *S. cerevisiae* cells were pre-loaded with 5 µg/ml dihydrorhodamine 123 for 15 min and then exposed to various concentrations of sodium arsenite, hydrogen peroxide or menadione. At 15, 30, 60 and 120 min time-points aliquots of cells were taken and immediately analyzed by flow cytometry to measure levels of green fluorescence of rhodamine 123 formed after oxidation of dihydrorhodamine 123 by ROS [70]. Untreated samples were used as a control of autofluorescence level.

### Measurements of DNA Oxidation

Genomic DNA was isolated from yeast cells treated with various concentrations of sodium arsenite, hydrogen peroxide and menadione for 2 h. Next, to obtain nucleosides DNA samples were digested with P1 nuclease at 37°C for 2 h and subsequently incubated with alkaline phosphatase at 37°C for 1 h. About 10 µg/ml of DNA were used to determine oxidative DNA damage in the form of 8-hydroxy-2′-deoxyguanosine using an ELISA-based kit (Cell Biolabs) according to the manufacturer's instructions.

### The Yeast Comet Assay

The alkaline comet assay was performed according to the protocol adopted for yeast cells [41]. Approximately 10^6^ cells from each treatment were harvested by centrifugation and mixed with 1.5% low melting agarose in S buffer (1 M sorbitol, 25 mM KH_2_PO_4_, pH 6.5) containing 2 mg/ml zymolyase (20T; 20 000 U/g). 200 µl of this mixture were spread over a slide coated with a water solution of 0.5% normal-melting agarose, covered with a cover slip and incubated for 45 min at 30°C for enzymatic degradation of yeast cell walls. To solidify the gel, the slides were kept at 4°C for 10 min after which the cover slips were removed. Slides were incubated in a lysis buffer (30 mM NaOH, 1 M NaCl, 0.05% laurylsarcosine, 50 mM EDTA, 10 mM Tris-HCl, pH 10) for 2 h at 4°C in order to lyse yeast spheroplasts. To remove the lysis solution, the slides were washed three times for 20 min at 4°C in an electrophoresis buffer (30 mM NaOH, 10 mM EDTA, 10 mM Tris-HCl, pH 10). The slides were then submitted to electrophoresis in the same buffer for 20 min at 25 V at room temperature. After electrophoresis, the slides were incubated in a neutralization buffer (10 mM Tris-HCl, pH 7.4) for 10 min, followed by consecutive 5 min incubation in 76% and 96% ethanol. The slides were then air-dried and visualized immediately or stored at 4°C for later observation. For visualization in a fluorescence microscope, the slides were stained with 2 µM YOYO-1 and 30 representative images of each slide were acquired at a magnification of ×400 using an Olympus BX61 fluorescence microscope. The images were analyzed with the help of Comet Assay IV image-analysis system software from Perspective Instruments to measure tail length (µm) and tail DNA (%). Tail moment (arbitrary unit) was calculated by multiplying the percentage of DNA in the tail by the distance between the center of mass of the comet head and the center of mass of the comet tail.

### Pulsed-Field Gel Electrophoresis

Preparation of agarose-embedded genomic DNA was performed with CHEF Genomic DNA Plug Kit (BioRad) following manufacturer's protocol. Briefly, 6×10^7^ cells was embedded in 100 µl of 0.75% low-melting agarose and incubated with lyticase for 2 h at 37°C. This was followed by digestion with proteinase K for overnight at 50°C. Plugs were washed 4 times for 1 h in a Wash buffer and stored in the same buffer at 4°C. The electrophoresis was performed using CHEF-DR III Pulsed Field Electrophoresis Systems (BioRad). The *S. cerevisiae* chromosome samples were resolved in 1% agarose at 6 V/cm for 22 h with a 60–120 s switch time ramp at 14°C. To separate the *S. pombe* chromosomes samples were resolved in 0.8% agarose at 1.5 V/cm for 72 h with a 1800 s switch time ramp at 14°C. Gels were stained with ethidium bromide (1 mg/ml) for 1 h and destained with 0.5× TBE buffer for 1 h and photographed.

### Southern Analysis

DNA separated with PFGE was transferred to a Hybond-N+ nylon membrane (GE Healthcare) by a capillary transfer and UV crosslinked. Next, membrane was hybridized with the 288 nt fragment of the *LEU2* gene (present both in the circular chromosome III as well as in the chromosome II) labeled with DIG High Prime DNA Labeling and Detection Starter Kit II (Roche) following manufacturer's protocol.

### Detection of DNA Repair Foci

To analyze formation of Rad52-YFP and Rfa1-YFP nuclear foci, live cells were observed with an Axio Imager M1 epifluorescence microscope (Carl Zeiss, Germany) equipped with a 100× oil immersion objective (Plan-Neofluar 100×/1.30), a GFP filter set and differential interference contrast (DIC). Images were collected using AxioCam MRc digital color camera and processed with AxioVision 4.5 software.

## Supporting Information

Table S1 Yeast strains used in this work.
(DOCX)
