# Supplementary material for: Oxidative Stress and Replication-Independent DNA Breakage Induced by Arsenic in Saccharomyces cerevisiae
Source: PLoS Genet. 2013 Jul 25;9(7):e1003640. doi: 10.1371/journal.pgen.1003640 (PMC3723488; doi:10.1371/journal.pgen.1003640)
Supplement: Table S1 — Yeast strains used in this work. (DOCX) [file pgen.1003640.s001.docx]

**Table S1.** Yeast strains used in this work.

| Strain | Description | Source |
| --- | --- | --- |
| *S. cerevisiae*: | | |
| W303-1A | *MAT***a** *ade2-1 can1-100 ura3-1 his3-11,15 leu2-3,112 trp1-1 RAD5* | R. Rothstein |
| RW105 | W303-1A, *acr3*Δ*:: loxP-kanMX-loxP ycf1*Δ*::loxP* | R. Wysocki |
| MC002 | W303-1A, *rad51*Δ*::kanMX6* | M. Cal-Bakowska |
| MC006 | W303-1A, *rad52*Δ*::kanMX6* | M. Cal-Bakowska |
| MC023 | W303-1A, *rad59*Δ*::kanMX6* | M. Cal-Bakowska |
| MC019 | W303-1A, *rad18*Δ*::kanMX6* | M. Cal-Bakowska |
| N1-2B | W303-1A, *tel1*Δ*::URA3* | N. Lowndes |
| N1-3A | W303-1A, *mec1-1* | N. Lowndes |
| IL001 | W303-1A, *mec1-1 tel1*Δ*::TRP1* | This study |
| IL002 | W303-1A, *rad9*Δ*::kanMX6* | This study |
| IL003 | W303-1A, *apn1*Δ*::TRP1 apn2*Δ*::kanMX6* | This study |
| IL004 | W303-1A, *rad14*Δ*::kanMX6* | This study |
| IL005 | W303-1A, *dnl4*Δ*::kanMX6* | This study |
| IL006 | W303-1A, *yku70*Δ*::kanMX6* | This study |
| IL007 | W303-1A, *yku70*Δ*::kanMX6 rad51*Δ*::TRP1* | This study |
| IL008 | W303-1A, *yku70*Δ*::kanMX6 rad59*Δ*::TRP1* | This study |
| IL009 | W303-1A, *rad59*Δ*::kanMX6 rad51*Δ*::natNT2* | This study |
| IL010 | W303-1A, *apn1*Δ*::TRP1 apn2*Δ*::kanMX6 rad51*Δ*::natNT2* | This study |
| W3749-14C | W303-1A*, ADE2* *bar1::LEU2 RAD52-YFP* | R. Rothstein |
| W3775-12C | W303-1A, *ADE2* *bar1::LEU2 RFA1-YFP* | R. Rothstein |
| MWJ49 | *MAT*α *leu2-3,112 ade5-1 his7-2 ura3Δ trp1-289* | M. Resnick |
| IL011 | MWJ49, *rad59*Δ*::natNT2* | This study |
| DB1033 | *MAT*α *ura3-52* | J. Brouwer |
| Y262 | *MAT*α *his4-539 ura3-52 rpb1-1* | J. Brouwer |
| *S*. *pombe*: |  |  |
| YA104 | *ura4-*Δ*18 leu1-32 his3-*Δ*1 arg3-*Δ*1 h+ mat1P*Δ*17::LEU2* | H. Iwasaki |
| YA223 | *rad52B::ura+ ura4-*Δ*18 leu1-32 ade6-M216 h- smt-0* | A. Pastnik |
| T3 | *rhp51::his3+ ura4-*Δ*18 leu1-32 his3-*Δ*1 arg3-*Δ*1 h- smt-0* | H. Iwasaki |
